# Distribution of FcγR gene polymorphisms among two sympatric populations in Mali: differing allele frequencies, associations with malariometric indices and implications for genetic susceptibility to malaria

**DOI:** 10.1186/s12936-015-1082-8

**Published:** 2016-01-19

**Authors:** Mariama Cherif, Daniel Amoako-Sakyi, Amagana Dolo, Jan-Olov Pearson, Ben Gyan, Dorcas Obiri-Yeboah, Issa Nebie, Sodiomon B. Sirima, Ogobara Doumbo, Marita Troye-Blomberg, Maiga Bakary

**Affiliations:** Centre National de Recherche et de Formation sur le Paludisme, Ouagadougou, Burkina Faso; Polytechnic University of Bobo Dioulasso, Bobo Dioulasso, Burkina Faso; Department of Microbiology and Immunology, College of Health and Allied Sciences, School of Medical Sciences, University of Cape Coast, Cape Coast, Ghana; Immunology Department, Noguchi Memorial Institute for Medical Research, University of Ghana, Legon, Accra, Ghana; Department of Epidemiology of Parasitic Diseases, Faculty of Medicine and Odonto-Stomatology, Malaria Research and Training Centre, USTTB, Bamako, Mali; Department of Molecular Biosciences, The Wenner-Gren Institute, Stockholm University, Stockholm, Sweden

## Abstract

**Background:**

Genetic polymorphisms in the complex gene cluster encoding human Fc-gamma receptors (FcγRs) may influence malaria susceptibility and pathogenesis. Studying genetic susceptibility to malaria is ideal among sympatric populations because the distribution of polymorphic genes among such populations can help in the identification malaria candidate genes. This study determined the distribution of three FcyRs single nucleotide polymorphisms (SNPs) (FcγRIIB-rs1050519, FcγRIIC-rs3933769 and FcγRIIIA-rs396991) among sympatric Fulani and Dogon children with uncomplicated malaria. The association of these SNPs with clinical, malariometric and immunological indices was also tested.

**Methods:**

This study involved 242 Fulani and Dogon volunteers from Mali age under 15 years. All SNPs were genotyped with predesigned TaqMan^®^ SNP Genotyping Assays. Genotypic and allelic distribution of SNPs was compared across ethnic groups using the Fisher exact test. Variations in clinical, malariometric and immunologic indices between groups were tested with Kruskal–Wallis H, Mann–Whitney U test and Fisher exact test where appropriate.

**Results:**

The study confirmed known malariometric and immunologic differences between sympatric Fulani and non-Fulani tribes. Parasite density was lower in the Fulani than the Dogon (p < 0.0001). The mutant allele of FcγRIIC (rs3933769) was found more frequently in the Fulani than the Dogon (p < 0.0001) while that of FcγRIIIA (rs396991) occurred less frequently in the Fulani than Dogon (p = 0.0043). The difference in the mutant allele frequency of FcγRIIB (rs1050519) between the two ethnic groups was however not statistically significant (p = 0.064). The mutant allele of rs396991 was associated with high malaria-specific IgG1 and IgG3 in the entire study population and Dogon tribe, p = 0.023 and 0.015, respectively. Parasite burden was lower in carriers of the FcγRIIC (rs3933769) mutant allele than non-carriers in the entire study population (p < 0.0001). Carriers of this allele harboured less than half the parasites found in non-carriers.

**Conclusion:**

Differences in the allelic frequencies of rs3933769 and rs396991 among Fulani and Dogon indirectly suggest that these SNPs may influence malaria susceptibility and pathogenesis in the study population. The high frequency of the FcγRIIC (rs3933769) mutant allele in the Fulani and its subsequent association with low parasite burden in the entire study population is noteworthy.

## Background

Variations in malaria susceptibility among populations living in endemic areas have been known for decades [1–3]. Sympatric populations, such as the Fulani and their sympatric neighbours, amply demonstrate this phenomenon. Epidemiologically, the Fulani have been consistently shown to be naturally less susceptible to malarial infections than their sympatric neighbours [2–8]. A clear biological explanation of the basis of this protection is still lacking. The observation of larger spleen sizes in the Fulani compared to their sympatric neighbours in the 1970s and 1980s suggested that the spleen may have a role in protecting the Fulani from malaria [5, 9]. Subsequent studies have provided clues to other possible biological mediators of this interethnic difference in malaria susceptibility. For instance, in Burkina Faso, the Fulani were shown to have lower parasite densities and stronger humoral immune responses to malaria antigens than their Mossi and Rimaibes sympatric neighbours [4, 6]. Similarly, a functional deficiency in T cell regulation is thought to contribute to the Fulani’s protection against malaria [10]. Several immuno-epidemiology studies have reported on the possible roles of different immune factors in this phenomenon and this has been extensively reviewed [11]. A review of the existing body of evidence suggests a significant role for immune-mediated mechanisms of protection; however, the immunogenetics of this protection are currently poorly explained.

Traditional host genetic factors known to influence malaria susceptibility have so far failed to adequately explain why the Fulani are less susceptible to malaria than their sympatric neighbours [12–15]. Recent malaria immunogenetic studies have suggested roles for a few genes but that notwithstanding, the immunogenes that mediate this protection are yet to be clearly identified [16, 17]. Research is ongoing to identify the immunogenes that may influence interethnic variability in malaria susceptibility. Of all the candidate genes, the Fc gamma receptors (FcγRs) for IgG are probably the most extensively studied. FcγRs for IgG are expressed on a variety of immune cells, including monocytes and other leucocytes. They typically bind to opsonized pathogens to activate a variety of cellular immune responses that may culminate in the control of an infection [18]. Thus, FcγRs are thought to be an important link between humoral and cellular immune response [18].

Studies on FcγRs polymorphisms and malaria susceptibility have so far focused on the FcγRIIa genotypes, which affect binding of different IgG sub-classes. The best known of the FcγRs polymorphisms is the FcγRIIa (H/R131), which has been associated with levels of anti-malarial IgG2 and IgG3 antibodies [19, 20]. Compared to their sympatric neighbours, the Fulani in Sudan were shown to have a higher frequency of the FcγRIIa-H131, which is associated with higher antibody levels [20]. This difference in allelic distribution was however not seen among the Fulani and Mossi in Burkina Faso [21]. Several other single nucleotide polymorphisms (SNPs) in FcγRs have been associated with malaria susceptibility but their contribution to interethnic difference in malaria susceptibility is unclear [22–25]. The genes encoding FcγRs are interesting and challenging to study for a number of reasons. Firstly, the individual genes (FCGR2A, FCγR2B, FCGR2C, FCGR3A, FCGR3B) in this cluster exhibit high homology between themselves [26]. For instance, FCGR2A and FCGR2B have approximately 95 % homology in their exons that code for their respective extracellular domains [27]. Secondly, FCGR2C, which encodes FcgRIIc, appears to be the product of an unequal crossover event between FCGR2A and FCGR2B [27]. Lastly, an apparent genotype-phenotype mismatch and the possibility that a SNP in one gene can affect the function of proteins encoded by a different gene in the FcγRs cluster is intriguing [27]. These features present formidable challenges to the study of this gene cluster and its polymorphisms, particularly their introns.

This study focused on three SNPs in the FcγRs gene cluster that are yet to be studied in relation to malaria: two intronic SNPs (FCGR2C-rs3933769 and FCGR3A-rs396991) and a functional SNP in the FCGR2B-rs1050519. The aim of this study was to determine the distribution of these SNPs among sympatric Fulani and Dogon tribes living in Mali with mild malaria. The study further investigated the association of these SNPs with various clinical, malariometric and immunological indices in children with mild malaria. The results of this study revealed differences in allelic frequencies in FC2R2C (rs3933769) and FCGR3A (rs396991) SNPs among the Fulani and Dogon in the study population. This finding indirectly suggests that the genes may contribute to the interethnic variability in malaria susceptibility seen among the Fulani and their sympatric neighbours.

## Methods

### Study participants

The study was conducted in the West African rural village of Mantéourou, about 850 km from Bamako, Mali. Fulani and Dogon tribes, who have distinct cultural and ethnic differences in spite of living within 0.5 km of each other, inhabit this village. The Fulani are cattle breeders while the Dogon are farmers. The Fulani and Dogon do not intermarry [7, 8]. Climatically, Mantéourou is a Sahelian area with dry seasons from October to May and a rainy season from June to October. The study comprised 242 volunteers under the age of 15 years belonging to the Fulani (26 %) and Dogon (74 %) tribes. Study volunteers were recruited through two cross-sectional surveys conducted at the end of the rainy season (October/November) and the dry season (March/April) of 2007. Volunteers were enrolled into the study through active malaria case search. Potential volunteers were clinically examined and their medical histories taken. Potential volunteers with mild malaria who also satisfied other inclusion criteria were recruited into the study after their parents/legal guardians had consented to their participation. Clinical and parasitological data were obtained from study volunteers.

### Malaria diagnosis, case definition and clinical information

Thick blood films were made and examined as described elsewhere [8]. Parasite densities were estimated using an assumed leucocyte count of 7500 leucocytes per microlitre of blood [7]. A slide was declared negative if no parasites were identified after 300 HPF (high-powered field) examination. Ten per cent of the slides, randomly selected by a physician, were double-read as a quality control measure. Clinical malaria was defined as the presence of fever (axillary temperature of at least 37.5 °C) plus the presence of *Plasmodium falciparum* parasites on the thick blood smear, in the absence of any other known illnesses. Exclusion criteria included: (1) any confirmed or suspected condition of immunosuppression by diseases, including HIV (no screening test has been done for this purpose) as determined by the physician; (2) chronic administration (defined as more than 14 days) of immunosuppressants or other immune-modifying drugs at the start of the study (this included oral steroids and inhaled steroids, but not topical steroids); (3) any chronic diseases (cardio-vascular, hepatic and renal) suspected by the physician to cause any supplementary risk to the volunteer; and, (4) any other circumstances and conditions suspected by the physician to be a risk for volunteer health. Axillary temperature of all volunteers was measured and their spleen size scored by Hackett’s method, dichotomized as enlarged or not enlarged.

### Genotyping

DNA was extracted from buffy coat using QIAamp DNA Blood Mini Kit (QIAgen, Inc.) following manufacturer’s protocol and stored at −20 °C. All SNPs were genotyped using TaqMan^®^ SNP Genotyping Assays following a wet DNA method described elsewhere [21]. In brief, PCR amplification was performed in 25 µl reactions using 2 µl of genomic DNA (10 ng/µl), 12.5 µl of 2× TaqMan Universal PCR Master Mix (No AmpErase UNG), 1.25 µl of 20× working stock of SNP Genotyping Assay, and 9.25 µl of DNA free water. The PCR was carried out in an Eppendorf Mastercycler (Eppendorf AG, Hamburg, Germany) using a 10-min enzyme activation at 95 °C followed by 40 cycles with 92 °C for 15 s and 60 °C for 1 min. Allelic discrimination was read and analysed by Applied Biosystems 7900HT Fast Real-Time PCR System using two TaqMan^®^ MGB probes for genotype detection.

### Measurement of antibodies

Serum levels of IgG, IgG1 and IgG3 antibodies were determined by standard indirect ELISA. Briefly, EIA/RIA plates (Costar, Corning, NY, USA) were absorbed overnight at 4 °C with 50 ml/well of crude percoll-enriched lysate of parasite-infected erythrocyte [F32 antigen] (10 mg/ml) diluted in sodium carbonate buffer (pH 9.6). The plates were blocked with 100 ml of sodium carbonate buffer diluted in 0.5 % bovine serum albumin (BSA) for 2 h at 37 °C. After four washings with ELISA washing buffer [PBS with Tween −20 and 0.15 % Kathon (Mabtech, Nacka, Sweden)] in a microplate washer (Skan washer 300, CA, USA), the plasma samples diluted in incubation buffer, phosphate buffered sulphate (PBS + 0.5 % BSA) 1:1000 (IgG) and 1:400 (IgG1 and IgG3) were added in duplicates and incubated for 1 h at 37 °C. Bound IgG were detected with goat anti-human IgG-ALP (1:2000) (Mabtech, Nacka, Sweden) while IgG sub-classes were detected with mouse anti-human; IgG1 1:1000 (SkyBio, Bedfordshire, UK), IgG3 1:1000 (Caltag Laboratories, Paisley, UK). ALP-conjugated to goat anti-mouse Ig (Dakopatts, Glostrup, Denmark) (1:1000) was used to detect IgG1, while Streptavidin-ALP (Mabtech) 1:2000 was added for IgG3 detection. The assays were developed with *p*-nitrophenyl phosphate (Sigma-Aldrich GmbH, Steinheim, Germany). The plates were read using molecular devices (Vmax^®^ Kinetic microplate reader, Menlo Park, USA) at 405 nm and analysed with SoftMax^®^ Pro software 5.2 molecular devices. Plasma samples collected earlier from Fulani individuals residing in Burkina Faso were randomly selected and used as the positive controls, and Swedish donors who had not experienced malaria and had never travelled to a malaria-endemic area were used as negative controls. Seropositivity was based on mean antibody levels + 2SD of ten non-malaria-exposed Swedish donors.

### Data analysis

Unless otherwise stated, all statistical analysis was implemented using Stata Statistical Software (Release 13, StataCorp LP, College Station, TX, USA). Demographic variables such as age and sex were compared across ethnic groups (Fulani and Dogon) using the Fisher exact test (χ^2^-test). Allelic distribution was tested for conformity with Hardy-Weinberg equilibrium (HWE) [28]. The genotypic and allelic distribution of SNPs studied was also compared across ethnic groups using the Fisher exact test. To gain insight into the malariometric and immunologic indices in the study population, the distribution of these indices was compared across ethnic groups using Mann–Whitney U test. To determine if the SNPs studied influence malariometric and immunologic indices, a codominant genetic model of inheritance was assumed. The effects of SNPs on malariometric and immunologic indices were tested using Mann–Whitney U test and Kruskal–Wallis analysis of ranks. A p <0.05 was deemed statistically significant.

### Ethical considerations

Informed consent was obtained from parents or legal guardians of study volunteers in a two-step procedure. First, oral community consent was obtained prior to the study, where the whole community was informed of the aim of the study. Secondly, individual consent was obtained at the time of blood collection, with all literate volunteers/guardians signing a consent form, and illiterate volunteers consented by fingerprint. The ethical committee of the National Ethics Committee in Mali approved the study.

## Results

### Characteristics of study population

The general characteristics of study volunteers are summarized in Table 1. A total of 242 children from Fulani (26 %) and Dogon (74 %) tribes participated in this study. Study participants included Fulani and Dogon males and female no older than 15 years. Median ages of participants from the two ethnic groups were comparable (p = 0.667) and so were the sex ratios (p = 0.39).

**Table 1:** General characteristics and clinical indices of study participants

	Fulani (n = 63)		Dogon (n = 179)		*P* value
	N [Median]	% (Range)	N (Median)	% (Range)	
Demography					
Age (in years)					
0–4	35	55.56	87	48.60	0.667
5–9	21	33.33	70	39.11	
10–15	7	11.11	22	12.29	
Sex					
Male	32	50.79	102	56.98	0.39
Female	31	49.21	77	43.02	
Clinical data					
Hb (g/dL)	[8.7]	(4.6–13.4)	[9.6]	(3.4–15.0)	0.022*
Parasite density (×25)	[119]	(2–2060)	[1000]	(8–10,120)	<0.0001*
Splenomegaly	32	56.14	56	31.46	<0.0001*
Fever prevalence	26	13.90	161	86.63	<0.0001*

Values in [ ] are median values

* Statistically significant differences (*p* < 0.05)

### Clinical and malariometric indices among the Fulani and Dogon

Haemoglobin (Hb), fever prevalence, parasite density, and spleen size were measured to enable comparison of malariometric and clinical indices among the Fulani and Dogon. Table 1 shows the clinical and malariometric indices among the study population. The Dogon were found to have a significantly higher Hb level than the Fulani (p = 0.022). The percentage of children with enlarged spleen was higher in the Fulani (56.14 %) than the Dogon (31.46 %) (p < 0.0001). Parasite density (p < 0.0001) and fever prevalence (p < 0.0001) were lower in the Fulani than the Dogon. Parasite density in the Dogon was almost 11-fold higher than that found in the Fulani (see Table 1). Apart from Hb levels, all other variable remained significantly different between Fulani and Dogon even after a Bonferroni correction reduced significant α level to ≈0.013. The difference in Hb levels between Fulani and Dogon (p = 0.022) approached significance.

### Anti-malaria specific antibody responses response among Fulani and Dogon

The levels IgG, IgG1 and IgG3 responses against F32 antigen were measured and compared between tribes to ascertain if they differed significantly. Table 2 shows the antibody levels between the Fulani and Dogon. The Fulani had stronger IgG and IgG1 responses than the Dogon (p < 0.0001 and p = 0.0013, respectively). Specific IgG3 responses were, however, comparable in the Fulani and the Dogon (p = 0.63). The difference in IgG and IgG1 levels between the Fulani and Dogon remained statistically significant after a Bonferroni correction reduced the significant α level to ≈0.017.

**Table 2:** IgG, IgG1 and IgG3 anti-malaria specific responses among the Fulani and Dogon

Antibodies	Fulani (n = 63)		Dogon (n = 179)		Fulani vs Dogon
	Median	Range	Median	Range	P value
IgG	10.39	(1.23–80.64)	5.13	(0.87–26.55)	<0.0001*
IgG1	34.0	(0.49–3084.5)	9.83	(0.001–67,204.7)	0.0013*
IgG3	0.35	(0.034–5.37)	0.33	(0–2.37)	0.63

* Statistically significant differences (*p* < 0.05)

### Genotypic and allelic distribution rs3933769, rs396991 and rs1050519 SNPs

In order to determine the genotypes and alleles that are over- or under-represented in the study population, the three SNPs were genotyped in the two tribes and their distributions compared. Table 3 shows the genotypic and allelic distribution of the three SNPs in the study population. Frequencies of the mutant alleles of FCGR2C (rs3933769), FCGR3A (rs396991) and FCGR2B (rs1050519) in the entire study population were 0.28, 0.23 and 0.16, respectively. The mutant allele of FCGR2C (rs3933769) occurred more frequently in the Fulani than the Dogon (p < 0.0001) while that of FCGR3A (rs396991) was found more frequently in the Dogon compared to the Fulani (p = 0.0043). The difference in the mutant allele frequency of FCGR2B (rs1050519) was however not statistically significant between the Fulani and Dogon tribes (p = 0.064). Apart from FCGR2B (rs1050519), which was not in HWE (p < 0.001), the other SNPs studied were in conformity with HWE: FCGR2C (rs3933769) (p > 0.05) and FCGRIIIA (rs396991) (p > 0.05) in the entire study population.

**Table 3:** Genetic and allelic distribution of SNPs among the Fulani and Dogon

SNP	Fulani (n = 63)		Dogon (n = 179)		P value
	N	%	N	%	
FcγRIIB (rs1050519)					
GG	41	65.08	139	77.65	
GT	16	25.40	32	17.88	
TT	6	9.52	8	4.47	
Frequency of mutant allele	22	34.92	40	22.35	0.064
FcγRIIC (rs3933769)					
CC	15	23.81	113	63.13	
CT	42	66.7	50	27.93	
TT	6	9.52	16	8.94	
Frequency of mutant allele	48	76.19	66	36.87	<0.0001*
FcγRIIIA (rs396991)					
AA	40	63.49	108	60.34	
AG	19	30.16	57	31.84	
GG	4	6.35	14	7.44	
Frequency of mutant allele	23	36.51	71	39.66	0.0043*

Genotypic and mutant allele distribution in Fulani, Dogon and entire study population

p Values are for mutant allele comparisons between Fulani and Dogon

* Statistically significant differences (*p* < 0.05)

### Association of SNPs with clinical and malariometric indices

In order to determine the SNPs that may contribute to malaria pathogenesis, the associations of SNPs with various malariometric and clinical indices was tested. A summary of SNP association with parasite density, Hb, spleen size, and fibril status is shown in Table 4. The rs3933769 SNP was associated with parasite density in the entire study population and carriers of the mutant allele harboured less than half of the parasite harboured by non-carriers (p < 0.0001). This association was not apparent within the Fulani (p = 0.235) and Dogon (p > 0.1) separately. The two other SNPs studied were not associated with parasite density (p = 0.064). None of the SNPs studied was associated with spleen size, Hb or fever prevalence.

**Table 4:** Association of SNPs with clinical and malariometric indices

SNP (gene)	Alleles		Comparison	Parasite density			Hb (g/dL)			Spleen Size					
				Fulani	Dogon	Fulani + Dogon	Fulani	Dogon	Fulani + Dogon	Fulani		Dogon		Fulani + Dogon	
										Normal	Enlarged	Normal	Enlarged	Normal	Enlarged
FCγRIIB (rs1050519)	Ref	G	GG vs TT/GT	100	1,030	665	8.75	9.6	9.3	56	65.63	78.69	75	74.8	71.59
	Alt	T		129.5	803.5	375	8.4	9.2	9.05	44	34.38	21.31	25	25.17	28.41
p Value				0.309	0.795	0.285	0.382	0.6	0.821	0.585		0.569		0.647	
FCγRIIC (rs3933769)	Ref	C	CC vs TT/CT	80	1,120	970	9.9	9.4	9.5	24	25	63.93	62.50	57.14	48.86
	Alt	T		140	709	386.5	8.5	9.65	8.95	76	75	36.07	37.50	42.86	51.14
p value				0.235	0.064	0.00001*	0.125	0.559	0.357	1.000		0.868		0.227	
FCγRIIIA (rs36991)	Ref	A	AA vs GG/AG	145.5	1000	675	8.7	9.5	9.2	72	62.5	58.2	66.07	60.54	64.77
	Alt	G		90	1000	511.5	8.5	9.5	9.3	28	37.5	41.8	33.93	39.46	35.23
p value				0.379	0.325	0.361	0.696	0.691	0.834	0.574		0.409		0.579	

Comparison of parasite density, Hb levels and spleen size in participants with wild and mutant alleles

Comparisons were done in the two tribes separately and in the entire study

* Statistically significant differences (*p* < 0.05)

### Association of SNPs with anti-malaria specific IgG, IgG1 and IgG3 antibodies

Humoral immune response is vital in malaria immunity and thus the association between SNPs and antibody responses was tested. Table 5 summarizes the association of SNPs with IgG, IgG1 and IgG3 anti-malaria specific antibodies. Apart from rs396991, which was associated with IgG1 (p = 0.023), none of the SNPs studied was associated with levels of antibody responses in the entire population. The rs396991 was neither associated with IgG1 within the Fulani (p = *0.127*) nor within the Dogon (p = *0.082*) populations. The rs396991 was also associated with IgG3 responses within the Dogon (p = 0.015) but no such association was seen within the Fulani group (p = 0.314).

**Table 5:** Association of SNPs with antibody levels

SNP (gene)	Alleles		Comparison	Antibody responses (Mean Log 10)								
				Fulani			Dogon			Fulani + Dogon		
				IgG	IgG1	IgG3	IgG	IgG1	IgG3	IgG	IgG1	IgG3
(FcγRIIB (rs1050519)	Ref	G	GG vs TT/GT	9.267	29.79	0.429	5.49	8.897	0.33	5.76	10.39	0.3475
	Alt	T		10.86	37.08	0.30	3.95	11.90	0.34	5.16	14.03	0.33
p Value				0.795	0.908	0.105	0.301	0.317	0.755	0.956	0.251	0.439
FCγRIIC (rs3933769)	Ref	C	CC vs TT/CT	14.61	44.31	0.33	5.35	10.21	0.35	5.71	11.31	0.34
	Alt	T		7.74	28.76	0.35	4.63	7.35	0.33	5.81	10.45	0.34
p Value				0.367	0.392	0.594	0.311	0.706	0.790	0.872	0.670	0.933
FCγRIIIA (rs36991)	Ref	A	AA vs GG/AG	9.83	12.27	0.35	5.09	6.66	0.32	5.74	8.31	0.34
	Alt	G		11.29	54	0.32	5.33	13.06	0.35	5.94	18.56	0.35
p Value				0.898	0.127	0.314	0.839	0.082	0.015*	0.712	0.023*	0.158

Comparison of antibody levels in participates with wild and mutant alleles

Comparisons were done in the two tribes separately and in the entire study

* Statistically significant differences (*p* < 0.05)

### Spleen size, haemoglobin levels, parasite density, and fever prevalence among study participants

To better understand the inter-relationships between the various malariometric indices, the associations between the spleen size, Hb, parasite density, and fever prevalence were tested (Table 6). The results showed that the study participants with enlarged spleen size had lower Hb (p = 0.032). Here again this association was seen in entire study population but not within the individual tribes. Spleen size was neither associated with parasite density (p > 0.1) nor fever prevalence (p > 0.1). Children who had fever had higher parasite density than those without fever (p < 0.0001).

**Table 6:** Relationship between various malariometric indices

Comparison	Fulani (n = 63)	Dogon (n = 179)	Fulani + Dogon (n = 242)
Spleen size/Hb	0.440	0.116	0.032*
Spleen size/parasitaemia	0.459	0.701	0.117
Spleen size/fever	0.834	0.767	0.229
Fever/parasite density	r = 0.140, p = 0.274	r = 0.256, p = 0.005*	r = 0.382, P < 0.001*

Mann–Whitney rank sum test was used to test all associations except the association between fever and parasite density, which was tested with Spearman’s correlation. Values in this Table are p values, except the comparison between fever and parasitaemia, where correlation coefficient is reported in addition to the p value

* Statistically significant differences (*p* < 0.05)

## Discussion

This study found that the allelic frequencies of rs396991 and rs3933769 were different between the Fulani and Dogon. This observation is noteworthy because of the confirmed and suggested roles of these SNPs. The rs396991 SNP represents a change from phenylalanine (F) to valine (V) at position 158 in the Fc fragment of IgG, low affinity IIIa, receptor (CD16a) gene [29]. The mutant allele of this SNP binds IgG1 and IgG3 with a higher affinity than the wild type, which binds fewer immune complexes thereby reducing the potential of inflammatory response [29, 30]. Functional mutation in the FCGR3A gene can potentially influence immune-mediated diseases since the FcγRIIIa receptors are found on several immune cells and constitute a vital link between humoral and cellular immunity. Thus, the finding that the rs396991 mutant allele occurs at higher frequency in the Dogon, who are more susceptible to malaria relative to Fulani, is alluring. The rs396991 has been shown to influence several immune-mediated diseases [31–34]. For instance, the homozygous wild genotype of rs396991 was under-represented in HIV-infected patients with Kaposi sarcoma [31]. This SNP is also suggested to play a limited role in rheumatoid arthritis, which is an immune-mediated disease [34]. The rs396991 is yet to be studied in relation to malaria but data from this study suggest that this SNP may play a role in malaria pathogenesis.

In line with previous studies, the Fulani had higher levels of anti-malaria specific IgG, IgG1 and IgG3 than the Dogon [19, 35]. However, this present study has expanded knowledge by further showing an association between rs396991 mutant allele and higher levels of IgG3 in the Dogon tribe. This is interesting considering the fact this SNP was associated with higher levels of IgG in Hutchison lymphoma patients [36]. The variable distribution of rs396991 among Fulani and Dogon, as well as its association with IgG, could be an indication of a possible role for this SNP in malaria susceptibility.

This study compared various malariometric indices among the sympatric tribes and, consistent with previous studies in The Gambia, Burkina Faso, Sudan and Mali [5, 6, 20], an enlarged spleen was more frequently seen among the Fulani compared to the Dogon. Also, consistent with previous studies is the finding of lower Hb levels, parasite density and fever prevalence in the Fulani relative to the Dogon [5, 15, 37]. Although these variations in malariometric indices among Fulani and non-Fulani sympatric neighbours have been known for decades, there is still no satisfactory explanation for some of them. For instance, the pronounced splenomegaly in the Fulani is poorly explained. A recent model explains that an innate propensity of the spleen to stringently retain parasitized red blood cells causes enlargement of the spleen, lowers Hb levels and reduces parasite density [38]. This present study showed an association between enlarged spleen and lower Hb levels but not parasite density. The data on spleen size, Hb levels, parasite density, and fever prevalence suggest that spleen size could be an important malariometric index.

Besides being more frequent in the Fulani than the Dogon, the intronic rs3933769 SNP of FCGR2C appeared to influence parasite density in the study population. Study participants with the mutant allele harboured fewer than half the parasites found in their counterparts with the ancestral allele. The FcγRIIc is suggested to play an important role in parasite clearance by effectively bridging humoral and cellular immunity [39]. This finding is particularly interesting considering that introns have been traditionally viewed as non-functional. Intronic SNPs have gained currency lately with the emergence of evidence on their possible functional roles [40, 41]. This finding adds to an increasing body of evidence that suggests roles for intronic SNPs. The intronic SNPs of FCγR2C are even more interesting considering that this gene exhibits a genotype-phenotype mismatch [27]. Heijden et al. recently found a novel intron 7 SNP of FCγRIIC that prevented some Caucasians with the FCγRIIC-ORF genotype from expressing FcγRIIb/c. Some individuals in the same population with the FCγRIIC-Stop genotype, which is thought to be a pseudogene, expressed the FcγRIIb/c receptor [27]. The extracellular domains of FcγRIIc and FcγRIIb are completely identical, a situation that further complicates the study of this gene cluster. Possibly several other SNPs or copy number variation (CNV) in FCγRIIC affects the expression of FcγRIIc on immune cells and influences their ability to induce antibody-dependent cellular cytotoxicity ADCC or alter the balance between activating and inhibitory signals [42]. Such polymorphisms may even affect the expression and function of other FcγRs than FcγRIIc [27]. The findings of this study leave room for speculating a role for rs3933769 in malaria pathogenesis and susceptibility. Further studies will be needed to make this SNP a *bona fide* malaria candidate polymorphism.

Some of the associations reported in this study were found in the entire study population and not in the two tribes separately. For instance, the associations between rs3933769 and parasite density, rs396991 and IgG1, enlarge spleen and lower Hb were all found in the entire study population only. These observations should be interpreted with care because even though the unequal numbers of Fulani and Dogon in this study do not necessarily pose a statistical problem, it could have reduced the power to detect statistical significance in two tribes separately.

## Conclusion

Taken together, this study confirms previous studies that showed interethnic difference between the Fulani and non-Fulani sympatric tribes with regard to clinical, malariometric and immunological indices. In the furtherance of knowledge, this study shows that the allelic distribution of two FCγR SNPs is different in the Fulani and Dogon living in sympatry in Mali. The study also shows that two of the SNPs are associated with parasite density and IgG1 and IgG3 responses. This study did not aim to determine the association between these FCγR SNPs and malaria. Further studies will be needed to determine the possible roles of these SNPs in malaria pathogenesis.

## Abbreviations

FcγR: Fc-gamma receptors
SNPs: single nucleotide polymorphisms
IgG: immunoglobulin G
IL: interleukin
PCR: polymerase chain reaction
ELISA: Enzyme linked immunosorbent assay
BSA: Bovine serum assay
PBS: phosphate buffered saline
HPF: high-powered field
HWE: Hardy–Weinberg equilibrium
RBC: red blood cell
ORF: open reading frame
CNV: copy number variation
ADCC: antibody-dependent cell-mediated cytotoxicity
DNA: deoxyribonucleic acid
